# Evidence of Positive Selection in Mitochondrial Complexes I and V of the African Elephant

**DOI:** 10.1371/journal.pone.0092587

**Published:** 2014-04-02

**Authors:** Tabitha M. Finch, Nan Zhao, Dmitry Korkin, Katy H. Frederick, Lori S. Eggert

**Affiliations:** 1 Division of Biological Sciences, University of Missouri, Columbia, Missouri, United States of America; 2 Department of Basic Sciences, College of Veterinary Medicine, Mississippi State University, Mississippi State, Mississippi, United States of America; 3 Informatics Institute and Department of Computer Science, University of Missouri, Columbia, Missouri, United States of America; Centre for Eye Research Australia, Australia

## Abstract

As species evolve, they become adapted to their local environments. Detecting the genetic signature of selection and connecting that to the phenotype of the organism, however, is challenging. Here we report using an integrative approach that combines DNA sequencing with structural biology analyses to assess the effect of selection on residues in the mitochondrial DNA of the two species of African elephants. We detected evidence of positive selection acting on residues in complexes I and V, and we used homology protein structure modeling to assess the effect of the biochemical properties of the selected residues on the enzyme structure. Given the role these enzymes play in oxidative phosphorylation, we propose that the selected residues may contribute to the metabolic adaptation of forest and savanna elephants to their unique habitats.

## Introduction

**Figure 1:**
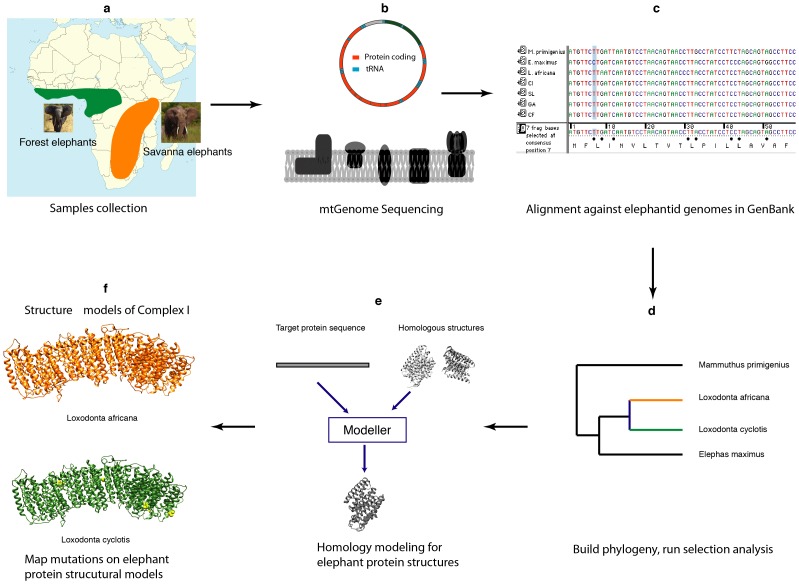
Flowchart outlining the methodological steps taken in our integrative approach to identify and analyze the structural biology of sites in the mitochondrial genome under positive selection in the African elephant. (a) Sample collection; green shows the range of the forest elephant (*L. cyclotis*) and orange shows the range of the savanna elephant (*L. africana*). (b) Sequencing the mtGenome; the protein coding genes encode for the subunits of the complexes involved in OXPHOS as shown in cartoon form. (c) Sequence alignment; complete mtGenome sequences for members of the Elephantidae were downloaded from GenBank, and to which we aligned our novel forest elephant sequences. (d) Phylogenetic and selection analyses; we inferred a phylogeny from our complete, aligned mtGenome sequence data and used the output to run analyses identifying sites that might be under positive selection. (e) Homology protein modeling; after identifying which genes (and complexes) might have sites under position selection, we searched the Protein Data Bank for homologous crystal structures, then input our elephant sequences and used Modeller to predict the elephant protein structures. (f) Mutation mapping; lastly, we mapped the residues that might be under positive selection onto our predicted elephant protein structures and assessed what impacts those substitutions found between *L. cyclotis* (green) and *L. africana* (orange) might have on the function of the protein in order to relate that to biological differences.

One of the central questions in molecular evolution revolves around whether natural selection at the DNA sequence level can be linked to adaptive phenotypic changes in the organism [1]. Genetic mutations in protein coding genes can affect the folding and 3-D structure of the protein produced, creating a cascade that may alter protein-protein interactions and modify biochemical pathways and cellular processes, all of which could affect the phenotype of the organism in a way that would impact its fitness [2]. Given the unique selective pressures of the environment in which an organism lives, those changes that confer fitness benefits may become fixed adaptations within a species over time. To elucidate the relationship between genetic variation and adaptive phenotypic traits, we adopted an integrative approach that combined detection of a molecular signature of selection with structural biological analyses to assess how the genetic changes affect the resulting protein and downstream networks that can be linked to adaptive phenotypic traits (Figure 1).

The mitochondrial genome (mtGenome) is an excellent system in which to study adaptive evolution. The 13 protein-coding genes in the mammalian mtGenome, along with dozens of nuclear genes, encode the protein subunits that make up four out of the five complexes of the electron transport chain (ETC) where the oxidative phosphorylation (OXPHOS) pathway occurs. OXPHOS plays a crucial role in energy metabolism and heat production, and through this pathway, mitochondria produce the majority of ATP that drives cellular processes. As a result, these proteins are under high functional constraint. However, given that metabolic requirements vary greatly across species, different selective pressures may be acting on these conserved complexes that lead to adaptive modifications.

The evolutionary history and phenotypic variation of the family Elephantidae make it an appropriate system for studying the adaptive evolution of the mtGenome in a long-lived, free-ranging mammal. The recent acquisition of whole mtGenomes for the extinct woolly mammoth (*Mammuthus primigenius*) and the American mastodon (*Mammut americanum*) have allowed for mitogenomic analyses of phylogenetic relationships among these taxa [3], [4]. The results of those studies suggest that the woolly mammoth and Asian elephant diverged shortly after diverging from their common ancestor with the African elephant. Mitogenomic and nuclear analyses of the taxonomy within *Loxodonta* suggest that the African savanna elephant (*Loxodonta africana*) and the African forest elephant (*Loxodonta cyclotis*) diverged approximately 5.5 million years ago [5], [6].

Ecological and morphological differences between African forest and savanna elephants result in differing metabolic requirements. African forest elephants are found in the tropical forest regions of West and Central Africa, and eat a diet largely of browse and fruits that includes a great diversity of plant species [7], [8]. In contrast, African savanna elephants are distributed in the savannas of eastern and southern Africa, and are generalist grazers/browsers that consume 60–95% of their forage as grasses [9], [10]. Additionally, forest and savanna elephants are morphologically distinct, with forest elephants having a substantially smaller body size than their savanna counterparts, shorter and rounder ears, and thinner, straighter tusks [11].

The selective neutrality assumption of mtDNA has been empirically tested and refuted across a broad range of organisms [12]. Recent studies have found evidence for molecular adaptations in the 13 protein-coding genes in the mtGenome [13], [14]. Some mutations have been associated with pathogenic disorders in humans and mice including exercise intolerance, neurological diseases and myopathy [15], [16], while others have been shown to have positive outcomes including greater aerobic energy metabolism [17]. In elephants and humans, Goodman et al. [18] show support for adaptively evolved mitochondrial functioning genes in the evolution of larger brain size and brain oxygen consumption. Considering the important role mitochondria play in metabolism, we might expect that some mutations in the mtDNA will result in ecological adaptations. When comparing the sequences of the protein-coding genes of the mtGenome across 41 mammal species, da Fonseca et al. [19] found great variation in the biochemical properties of amino acids at functional sites, concluding that these changes may be adaptive to the special metabolic requirements across the diverse taxa. Research on anthropoid primates found an accelerated rate of non-synonymous substitutions in mtDNA that are linked to phenotypic changes, such as an enlarged neocortex and extended lifespan [20]. Most recently, research on Pacific salmon (genus *Onchorhynchus*) identified multiple sites within mitochondrial genes that were under positive selection and examined those sites in a structural context based on crystallized bacterial protein complexes [21].

The five enzyme complexes of the OXPHOS pathway are embedded within the inner mitochondrial membrane. Four of these complexes contain varying numbers of mitochondrial encoded subunits in their structure. Complex I includes the seven subunits encoded by the NADH dehydrogenase (ND) genes (*ND1*, *2*, *3*, *4*, *4L*, *5*, *6*), the cytochrome *b* (*CYTB*) subunit is found in complex III, complex IV contains the three cytochrome oxidase (COX) gene subunits (*COXI*, *COXII*, *COXIII*), and lastly, the ATP synthase 6 (*ATP6*) and ATP synthase 8 (*ATP8*) subunits make up part of complex V. As electrons are passed through complexes I–IV, a proton-motive force is created to drive the synthesis of ATP from ADP and inorganic phosphate [22].

Knowing the native state of a protein allows for a more powerful analysis of the biochemical properties that may affect the structure and, ultimately, the function of that molecule. Homology protein structure modeling is a useful tool that involves taking the known 3-D structure of a closely related protein and using it as a template to model an unknown protein structure [23]. Because changes in the protein sequence can produce changes in the 3-D shape, the objective of this study was to investigate adaptive changes within African elephants by identifying regions of the mtGenome that may be under positive selection and to use homology protein structure modeling to assess whether these changes may alter the structure or function of the protein. This is the first study to take an integrated approach using selection analyses and structural biology to predict 3-D structures of the OXPHOS proteins for the African elephant to identify adaptive sites in the mtGenome (Figure 1). Furthermore, we are the first to look for evidence of positive selection between the African forest and savanna elephant. Previous work has focused solely on the savanna elephant, but we utilize the most complete dataset of available forest elephant mtGenome sequences, including two individuals sequenced from dung samples. As such, we provide a framework by which studies on adaptive evolution can be undertaken on free-ranging wildlife species that may be more easily studied through noninvasive sampling techniques.

## Results

### Sequence and Phylogenetic Analyses

**Figure 2:**
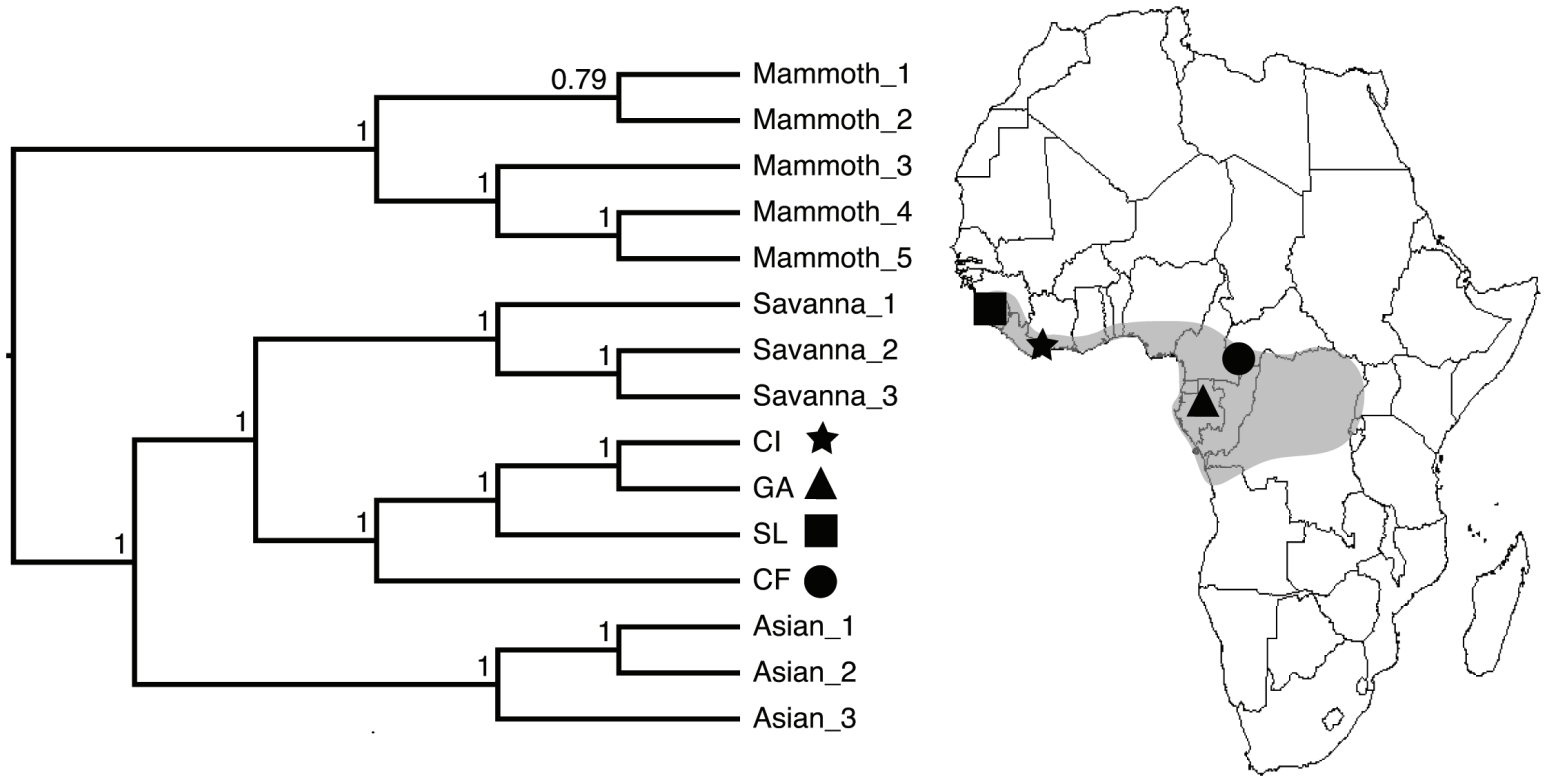
Whole mtGenome phylogeny for samples included in this study. Results from MrBayes are presented (PhyML shows same topology; 15,400 bp, 15 partitions) alongside a map of Africa showing the origin for the forest elephant samples (shaded area represents present-day forest zone). The star represents Taï National Park, Cote d'Ivoire (CI); triangle represents Lopé National Park, Gabon (GA); square represents Sierra Leone (SL); and circle represents Dzanga Sangha Forest Reserve, Central African Republic (CF).

We sequenced 16,030 bp of the mitochondrial genome from a West African forest elephant (Acc # KJ557424) and 16,030 bp from a Central African forest elephant (Acc # KJ557423). Start and stop codons in the forest elephant samples for each of the 13 protein coding genes are shared with those of the reference savanna elephant mtGenome (Acc # AB443879.1) [24]. The only sequence anomaly, also noted by Brandt et al. [5], is a 2 bp insertion in the *12S rRNA* gene for the Central African forest elephant that is not found in other elephantid mtGenomes. Relationships within the Elephantidae using the complete mtGenome are depicted in Figure 2. Excluding the clade of mammoths, the posterior probability for each clade is 1. In addition to the monophyly of *Loxodonta*, our findings confirm the deep divergence between African forest and savanna elephants [5]. This is the first study to sequence the entire forest elephant mtGenome from dung samples. This serves as a proof of concept for future research in this area that aims to focus on noninvasive sampling of free-ranging wildlife species that may be of conservation concern.

### Adaptive Evolution Analysis

Analysis in TreeSAAP identified several significant amino acid changes. Those that differ between forest and savanna elephants are found in complexes I and V of the ETC. In complex I, we found six significant changes between forest and savanna elephants in the *ND1*, *ND4*, *ND5* and *ND6* genes, and two in the *ATP6* gene of complex V (Table 1). The three individual savanna elephant samples included in this study all shared the same residue at each of the eight significant changes, whereas the four individual forest elephant samples show greater variation (Table 1). We focused further analyses on complexes I and V.

**Table 1:** Significant amino acid changes in the mitogenome identified by TreeSAAP.

Complex	Gene/Position	*Mammuthus primigenius*	*Elephas maximus*	*Loxodonta africana*	*Loxodonta cyclotis*:			
					CI	GA	CF	SL
I	ND1, 49	I	I	I	I	I	V	I
I	ND4, 15	A	A	A	T	T	V	T
I	ND5, 20	T	T	I	T	T	T	T
I	ND5, 21	T	L	T	I	T	I	A
I	ND6, 43	I	I	I	V	V	V	I
I	ND6, 45	S	S	G	S	S	S	S
V	ATP6, 7	A	A	T	A	A	A	A
V	ATP6, 10	D	D	Y	D	D	D	D

CF =  Central African Republic; CI =  Cote d'Ivoire; GA =  Gabon; SL =  Sierra Leona.

Both complexes I and V contain domains in the inner mitochondrial membrane. It is very challenging to solve tertiary structures of transmembrane proteins, but two homologous bacterial structures were found in the Protein Data Bank (PDB) [25] for complex I: one for *Thermus thermophilus* (PDB ID: 4HE8) [26] and the other for *Escherichia coli* (PDB ID: 3RKO) [27]. No high resolution homologous structures were found for complex V. Therefore, we proceeded with homology modeling analyses for complex I, but not for complex V.

### Complex I Structure and Function

Complex I is the first and largest enzyme complex in the OXPHOS pathway, and mutations in its subunits have been linked to many human neurodegenerative diseases [15]. This complex is known to be one of the largest membrane protein assemblies with 44 subunits comprising the eukaryotic complex, 14 of which are homologous to bacterial subunits and provide a catalytic core of the enzyme [28], [29]. It catalyzes the reactions that synthesize ATP by creating an electrochemical proton gradient. First, NADH is oxidized in the mitochondrial matrix, which provides two electrons to be transferred to quinone in the inner mitochondrial membrane [30]. This electron transfer is coupled with pumping four protons across the inner mitochondrial membrane, thus producing an electrochemical gradient. While no crystal structure of complex I from a multicellular eukaryote has been obtained, images from low-resolution electron microscopy have revealed that the eukaryotic complex I forms an L-shaped structure with a membrane arm embedded within the inner mitochondrial membrane and a peripheral hydrophilic arm that protrudes into the mitochondrial matrix [31].

Complex I is encoded by both nuclear and mitochondrial genes. The membrane domain in *T. thermophilus* confirms that the homologous eukaryotic subunits encoded by mtDNA genes *ND1*, *ND2*, *ND3*, *ND4*, *ND4L*, *ND5*, and *ND6*, are found in the membrane arm [26]. Similar results have been shown for the *E. coli* complex I structure, although the homologous subunit encoded by *ND1* was not crystallized because it readily dissociates from the complex [27]. It is believed that the coupling mechanism, by which the electrochemical gradient is created, occurs due to long-range conformational changes. Baradaran et al. [26] propose that the quinone-binding site is found at the interface of subunit *ND1* and the hydrophilic arm. Subunits *ND1*, *ND6* and *ND4L* form a proton-translocation channel that ejects a proton into the periplasm. During each cycle, three additional protons are transferred into the periplasm by proton pumps encoded by subunits *ND2*, *ND4* and *ND5*. Subunit *ND3* is thought to intertwine with *ND1* in order to stabilize the interface between the membrane and hydrophilic domains.

### African Elephant Complex I Structure

**Figure 3:**
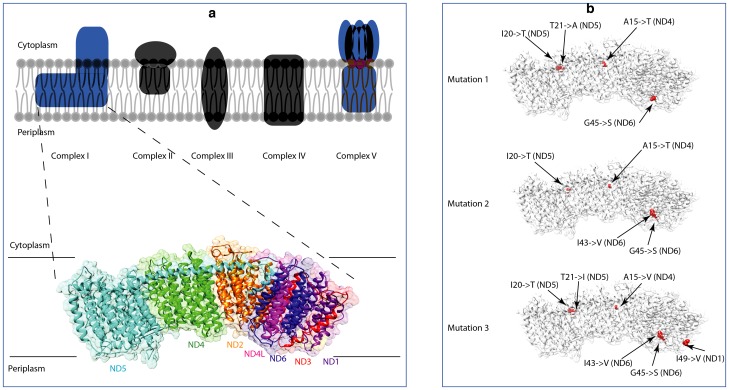
Our predicted models of the African elephant complex I. (a) Simplified drawing of the mammalian ETC with the five complexes that are involved in the OXPHOS pathway. These complexes are located on the inner mitochondrial membrane. The enlarged image shows the predicted African elephant protein structure for the mitochondrial DNA encoded genes of complex I. Chains are represented by different colors (dark purple  = *ND1*, orange  = *ND2*, red  = *ND3*, green  = *ND4*, light purple  = *ND4L*, light blue  = *ND5*, dark blue  = *ND6*). (b) The three different forest elephant mutation models. Selected amino acid substitutions are mapped onto the savanna elephant predicted structure and are shown in red. The Mutation 1 model represents SL, mutation 2 represents CI and GA, and mutation 3 represents CF. The mutations are labeled based on their chain ID, and with the savanna elephant residue listed before the altered forest elephant residue.

After homology modeling and side chain refinement, free loops that were not aligned with either of the two template structures were omitted, resulting in the final tertiary structure model for the African elephant complex I shown in Figure 3a. Root-mean-square deviation (RMSD) values and a TM-score were calculated as a quality assessment of the structure. As a comparison, a RMSD of 3.39 Å was found for 1,814 amino acid residues on the aligned chains (N, A, M, K, L, J) of the *T. thermophilus* and *E. coli* templates, and the TM-score between these two structures was 0.881. The 1,546 residue alignment of the savanna elephant structure with that for *T. thermophilus* resulted in a RMSD of 7.31 Å and a TM-score of 0.596, while the RMSD for the 1,371 residue alignment with *E. coli's* structure produced a value of 6.61 Å and a TM-score of 0.592. Considering the large size of the structure and the RMSD value between the two bacterial templates, the RMSD values for the elephant model demonstrate support for our predicted structure, as do our TM-scores, which are all greater than 0.5.

**Figure 4:**
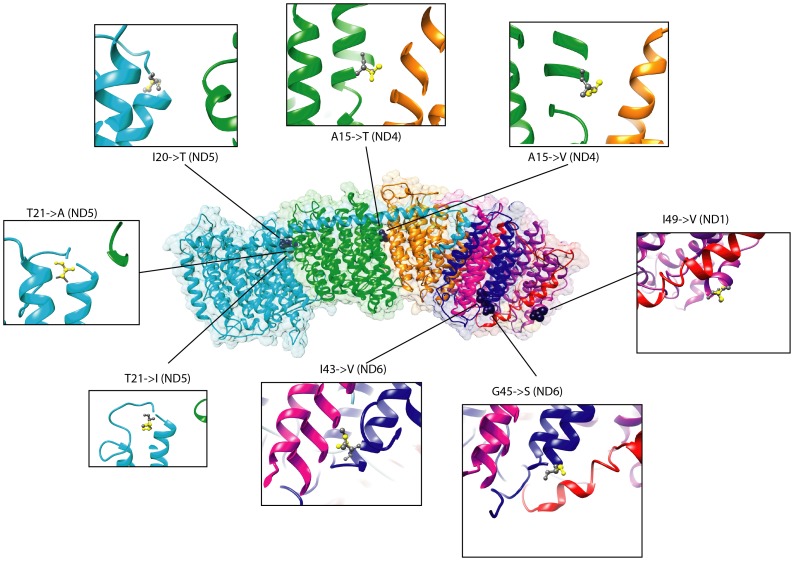
The atomic level structure for each of the selected amino acid substitutions as shown on our predicted model for the African elephant complex I. Mutations are shown in blue. The enlarged images show the African savanna elephant amino acid side chain in grey and the African forest elephant amino acid side chain in yellow.

For the four forest elephant samples included in this study, there are three possible combinations of mutations that are mapped onto our African elephant complex I structure (Figure 3b). Figure 4 shows the atomic structure for each of those mutations. To estimate whether the selected residue was buried inside the protein or on the surface, we calculated relative accessible surface areas (ASA) for each of the mutations. We applied a 5% threshold on accessibility to define whether a residue was found on the surface or was buried [32]. As such, we found that three of the mutation locations (*ND1,49, ND5,20*, and *ND6,45*) had values higher than 5% and are on the surface of the protein (Table 2). Four of the mutation locations have at least one chain-chain binding site for the mtDNA encoded subunits. It is possible these residues could interact with the nuclear encoded subunits that have not been sequenced.

**Table 2:** Interaction of residues with other protein subunits of Complex I as determined by the predicted African elephant protein structure.

Mutation	Binding Residue	Interacting Subunit(s)	Relative Accessible Surface Area (%)
ND1, 49	+	ND2, ND3	30.1
ND4, 15	+	ND2	0.2
ND5, 20	−	−	42.2
ND5, 21	−	−	0.8
ND6, 43	+	ND2, ND3, ND4L	0.1
ND6, 45	+	ND2, ND4L	54.0

### Structural and Functional Effects of Selected Residues in Complex I

The alignment of homologous structures for complex I reveals that each of the six significant mutations found in this study are in regions that are not highly conserved across species [26], [27], [33]. Based on the alignment of our *L. africana* complex I structure with that of *T. thermophilus*, we determined the location of our selected residues within the protein chains. Chain *ND1* has 9 transmembrane (TM) helices. The mutation at *ND1, 49* is located in TM1, which creates part of a narrow entryway for the quinone. Here, the forest elephant sample from CF has a valine while all other forest and savanna samples share an isoleucine. According to the Taylor classification [34], both of these amino acids are aliphatic, hydrophobic residues, so we would not expect this substitution to result in large structural changes. However, given its location near the quinone-binding site and because it is predicted to be both a surface residue and interact with subunits *ND2* and *ND3*, this may affect the overall conformation and/or efficiency of the entry point for the quinone molecule. Near subunit *ND1* and forming part of the fourth proton-translocation channel are two significant substitutions located at binding sites on subunit *ND6*, which contains five TM helices. At *ND6, 43*, located in TM2, savanna elephants along with the forest elephant SL sample display isoleucine whereas the other three forest elephants sampled have a valine. As described above, isoleucine and valine share similar biochemical properties. This site interacts with residues on three other chains encoded by *ND2*, *ND3*, *ND4L*, thus making it more likely to impact the overall structure of the proton-translocation channel *ND6* forms with subunits *ND1* and *ND4L*. Savanna elephants share a glycine at *ND6, 45*, which is found in the loop region between TM2 and TM3, while all forest elephant samples have a serine. Both of these amino acids are small, but serine is a polar residue and glycine is hydrophobic. This buried residue is at a protein binding site for chains *ND2* and *ND4L*, which may cause conformational changes for the proton-translocation channel and affect its efficiency. The remaining three substitutions are part of the membrane-bound proton pumps. Of the 14 TM helices in subunit *ND4*, position 15 is located in TM1 where it was found that savanna elephants have alanine while forest elephants from CI, GA and SL share a threonine residue and the sample from CF has valine. All three of these residues are small, but alanine is non-polar and slightly hydrophobic, valine is aliphatic and more hydrophobic, and threonine can be both polar and hydrophobic. This substitution is found on a binding site for another of the proton pumps encoded by gene *ND2* and forms part of a lipid-facing layer. Subunit *ND5* has significant substitutions at positions 20 and 21, both of which are found in TM1 (there are 16 total) that is also part of the lipid-facing layer. At residue 20, savanna elephant samples share an isoleucine and all forest elephant samples have threonine. Both residues can be hydrophobic with isoleucine classified as aliphatic and threonine also being polar. Lastly, at site 21, all savanna elephants and forest samples from CI and GA have threonine, while the SL forest elephant sample has alanine and CF has isoleucine. As previously described, isoleucine is the most hydrophobic residue and is also aliphatic, while alanine is less hydrophobic and polar, and threonine is polar. Although the amino acid substitutions observed between forest and savanna elephants at the proton pumps are not that unlike in their biochemistries, they are at locations that could alter the efficiency of the pumps, thus affecting the OXPHOS pathway and resulting in phenotypic changes between species. Mutations that affect a protein's interaction with other proteins that form a biochemical pathway are capable of altering the phenotype [2]. Four out of six of our selected mutations (Table 2) are at protein-binding sites and are likely affect the OXPHOS pathway of forest and savanna elephant species.

### Complex V Analyses

Complex V, or ATP synthase, was the other enzyme in OXPHOS where we identified significant amino acid changes between the forest and savanna elephant. The role of ATP synthase in OXPHOS is to phosphorylate ADP to synthesize an ATP molecule. ATP synthase is composed of two distinct units: the water soluble F1 portion that contains the catalytic sites and the transmembrane F0 portion that acts as a proton turbine [35].

We found two significant sites in the *ATP6* gene, which codes for subunit *a*, that is thought to participate directly in the proton flow [35]. Because of the difficulty in crystallizing membrane proteins, little information is known about the structure of the F0 proton channel [36] and therefore we have not conducted further structural analyses. We can, however, look at the biochemical differences for the residues of interest. At site seven of the *ATP6* gene *L. africana* has a threonine while all *L. cyclotis* samples have an alanine. Both are small residues, but threonine is polar and alanine is non-polar. Perhaps the greatest biochemical difference between amino acid substitutions is found on *ATP6* site 10 where savanna elephants share a tyrosine and the forest elephants have aspartic acid. Tyrosine has an aromatic side chain, is slightly hydrophobic and polar, while aspartic acid is also polar, but has a negative charge. In this complex, large conformational changes are required to occur in order to couple the passage of protons with the production of ATP. As a result, the selected amino acid substitutions between forest and savanna elephants could affect these conformational changes and alter the efficiency of ATP production, and thus metabolism, in these two species.

## Discussion

The 13 protein-coding genes of the mtGenome code for the machinery that make up the complexes of the ETC, which is a key biochemical pathway involved in the production of ATP and consequently is closely linked to metabolic activity. The objective of this study was to compare mtGenome sequences between the African forest and savanna elephant in order to identify sites in the mtGenome that might be under positive selection and to assess how those substitutions could result in adaptive differences between these two species. To accomplish this, we used an integrative approach that combined sequencing and structural genomic techniques to provide insights on how the selected residues might affect protein structure and likely function of the OXPHOS pathway.

Our results are in line with other studies that have found evidence of adaptive evolution in the ETC complexes [19], [21], [37]. Garvin et al. [21] detected a strong signal of positive selection in the *ND2* and *ND5* genes between species of Pacific salmon. Specifically, they linked the significant sites on the *ND5* gene to the structural piston arm of a proton pump and suggest the possibility that changes in the proton pump may have influenced fitness during the evolution of the salmon species studied. In an analysis of 41 mammalian species, da Fonseca et al. [19] found evidence of positive selection in the three proton pumps encoded by genes *ND2*, *ND4* and *ND5*. Research studies on equids argue that mutation patterns in the *ND6* gene are indicative of an adaptation to high altitude [37], [38].

While residues are conserved amongst *L. africana*, we see variability in the residues found in *L. cyclotis*. This finding might be expected given the higher genetic diversity known to occur in forest elephants [6]. Phylogeographic studies of forest elephants using mitochondrial DNA suggest that their evolutionary history is more complex than that of their savanna counterparts [39], [40]. A similar study on killer whales (*Orcinus orca*) found evidence of positive selection in the *CYTB* gene between two distinct ecotypes, and suggests these amino acid substitutions are ecological adaptations [41]. In addition, empirical research on sympatric haplotypes of *Drosophila simulans* suggest that mtDNA variation is responsible for phenotypic differences that include cold tolerance, starvation resistance and greater egg size and fecundity [42]. The varying selective pressures acting on populations of the same species under differing environmental conditions may lead to specialized metabolic adaptations in the mitochondrial genes that code for the OXPHOS pathway that functions to synthesize ATP and generate heat to maintain body temperature.

The morphological and ecological differences between the forest and savanna elephant could influence their respective metabolic requirements. Standard metabolic rate is a good descriptor for the minimal rate of energy flow for an animal. Based on the empirically tested equations for standard metabolic rate, it has been shown that, in general, larger organisms respire at a higher rate than smaller organisms [43]. Forest elephants have a more compact body stature than their savanna counterparts with one population comparison finding *L. cyclotis* to be 35–40% shorter than *L. africana* [44], thus, they consume less oxygen. One study on leukaemic cells linked mutations in the *ND1* gene to increased levels of oxygen consumption [45]. Other research on elephants found support for adaptive evolution in OXPHOS proteins that were related to higher brain oxygen consumption in these large animals[18]. In light of this previous work and our results on the selected amino acid substitutions between *Loxodonta* species, further investigations of the role phenotypic differences play in oxygen production and consumption are needed.

In addition, thermoregulation plays an important role in the biology and adaptation of the African elephant. As with standard metabolic rate, metabolic heat production scales with biomass where larger mammals have lower body temperatures [46]. Larger animals also have smaller surface area: volume ratios, resulting in less area available for heat transfer [47]. This physiological constraint is compacted even further for the savanna elephant given it inhabits hot, arid environments where seasonality causes extreme fluctuations in water and food availability. The forest elephant, however, experiences less dramatic inter-seasonal variation in its tropical closed-canopy forest habitat. Given the role of the OXPHOS pathway to generate heat and maintain body temperature, residue substitutions that reduce the coupling efficiency of ATP synthase would result in lower ATP production and increase heat production [48]. We found two mutations in complex V between forest and savanna elephants that may be under positive selection. Future work on crystallizing the ATP synthase enzyme will be needed to model this complex and map selected mutations to assess how they might affect the overall structure and function. Studies will also be needed to determine whether the mutations we detected may affect regulation via posttranslational modification [49].

While there are limitations to this study, we provide a framework for assessing the effects of selected amino acid substitutions on the structure of the OXPHOS pathway in non-model species. When working with free-ranging wildlife of conservation concern, it is often impractical and unethical to conduct empirical studies. We show that it is possible to collect noninvasive field sample to carry out meaningful selection and structural biology analyses. While we are limited in our capacity to test for the impact certain mutations have on physiology and function, we believe the changes we found in the mitochondrial genome for forest and savanna elephants play a role in their adaptive evolution.

We have taken a novel approach to studying the adaptive evolution of the mtGenome by combining phylogenetic and protein prediction methods to better understand the structural biology of the OXPHOS pathway in the African elephant. This is the first study to predict the protein structure from any of the ETC complexes for a specific study species to more accurately identify the locations of our selected residues. Given the lack of a high resolution structure for complex V, we were unable to use computational biology tools to predict the homologous structure for the African elephant. Nonetheless, our results provide evidence for sites that are under positive selection, which should be investigated further to assess the physiological impact these mutations have on metabolically-related life-history traits of African forest and savanna elephants. Future work includes sequencing the nuclear genes that code for protein subunits that complete the machinery for the OXPHOS enzyme complexes to better understand the protein interactions and how they might lead to functional changes between the species. Additionally, we aim to sequence samples spanning the range of *Loxodonta* to identify associations between adaptive changes and landscape features, such as in Foote et al.'s work [41], as well as phylogeographic patterns.

## Materials and Methods

### Ethics Statement

Samples for this study were collected noninvasively.

### Samples

Dung samples from unrelated African forest elephants were originally collected at Taï National Park, Cote d'Ivoire (CI), and Lopé National Park, Gabon (GA) as part of population level studies [39], [50]. We selected one sample from each park to sequence, therefore giving us two novel forest elephant mtGenome sequences for our study. These locations are deep within the forest zones of West and Central Africa, thus avoiding regions in which historical or contemporary hybridization may have occurred between forest and savanna elephants [51]. Approximately 20 g of dung were collected and boiled in the collection tube to prevent the transportation of pathogens, then stored in Queens College preservation buffer (20% DMSO, 0.25 M EDTA, 100 mM Tris, pH 7.5, saturated with NaCl [52]). Total genomic DNA was extracted from dung samples in a lab dedicated to noninvasive DNA extractions [53] using the Qiagen QIAmp DNA Stool Mini Kit (Qiagen, Valenica, CA, USA) with modifications as described in Archie [54]. In addition, we used previously published whole mtGenome sequences for members of the Elephantidae (Table 3).

**Table 3:** Accession numbers for samples included in this study.

Species	Sample Name	Accession #	Citation
*Mammuthus primigenius*	Mammoth 1	AP008987	Ozawa et al. unpublished
	Mammoth 2	DQ316067	Rogaev et al. 2006
	Mammoth 3	EU155210	Gilbert et al. 2008
	Mammoth 4	NC_007596	Krause et al. 2006
	Mammoth 5	DQ188829	Krause et al. 2006
*Elephas maximus*	Asian 1	DQ316068	Rogaev et al. 2006
	Asian 2	NC_005129	Rogaev et al. 2006
	Asian 3	EF588275	Maikaew et al. unpublished
*Loxodonta africana*	Savanna 1	AB443879.1	Murata et al. 2009
	Savanna 2	NC_000934.1	Hauf et al. 2000
	Savanna 3	DQ316069.1	Rogaev et al. 2006
*Loxodonta cyclotis*	CI	KJ557424	This study
	GA	KJ557423	This study
	SL	JN673264	Brandt et al. 2012
	CF	JN673263	Brandt et al. 2012

### DNA Amplification and Sequencing

We designed 44 primer pairs using a savanna elephant sequence (Acc # AB443879.1) as a template. Fragment sizes varied between 175 and 522 bp, and covered the entire mitochondrial genome excluding a variable number of tandem repeats (VNTR) found in the control region (Table S1). To sequence both ends of the VNTR, we amplified and cloned a 136 bp fragment using a Topo TA Cloning Kit (Invitrogen, Carlsbad, CA, USA). Ten clones per forest elephant sample were purified using the QIAprep Spin Miniprep Kit (Qiagen) and sequenced at the University of Missouri's DNA CORE in a 3730 DNA Analyzer (Applied Biosystems, Foster City, CA, USA). For all other fragments PCR was performed using an Eppendorf Mastercycler ep thermocycler in 25 μL volumes containing 1XPCR gold buffer, 0.2 μM dNTP, 0.5 U AmpliTaq Gold DNA Polymerase (Applied Biosystems), 1.5 mM MgCl_2_, 10XBSA (New England Bioloabs, Ipswich, MA, USA), 0.4 μM forward primer, 0.4 μM reverse primer, and 2 μL of DNA template. The profile included an initial denaturation step at 95°C for 10 minutes, followed by 50 cycles of denaturation at 95°C for 1 minute, annealing at 58°C for 1 minute, and primer extension at 72°C for 1 minute, ending with an elongation step at 72°C for 10 minutes. A negative control sample was included with every PCR to detect contamination of reagents. Amplification products were visualized in a 2% agarose gel and fragments of the correct length were purified with a QiaQuick PCR purification kit (Qiagen) and sequenced on a 3730xl 96-capillary DNA Analyzer (Applied Biosystems, Foster City, CA, USA) at the University of Missouri's DNA CORE facility.

### Phylogenetic Analyses

Sequences were assembled and aligned using Sequencher v. 4.5 (GeneCodes, Ann Arbor, MI). As nuclear insertions of mtDNA (numts) [55] are commonly found in elephant DNA extracted from hair samples [56], we examined the translation of all protein coding sequences to verify the open reading frame. We aligned both our novel forest elephant sequences to five mammoth, three Asian elephant, three savanna elephant and two additional forest elephant whole mtGenome sequences available in GenBank [57], thus bringing the dataset to 15 individuals (Table 3). The sequences included in this study are likely from unrelated individuals given that they were largely sampled from different countries. The mammoth was selected as an outgroup for phylogenetic analyses.

After inferring phylogenetic relationships using each of the 13 protein coding genes (*ATP6*, *ATP8*, *COX1*, *COX2*, *COX3*, *CYTB*, *ND1*, *ND2*, *ND3*, *ND4*, *ND4L*, *ND5*, *ND6*), we ran a concatenated data set with 15 partitions: each of the 13 protein coding genes, all tRNAs, and both rRNAs. Since using a single model of evolution for the entire mtDNA sequence may result in error, we selected a model of evolution for each partition using FindModel (Table S2) [58]. When certain samples (typically mammoth) had more amino acids than other taxa, protein coding gene alignments were edited to be the same length. To infer phylogenetic relationships among the 15 sequences, Bayesian inference with Markov chain Monte Carlo (MCMC) sampling was conducted using MrBayes v. 3.1 [59], [60]. The combined total alignment for the partitioned dataset was 15,354 bases including a 2 bp insertion in the 12S rRNA gene for the forest elephant samples from Gabon and the Central African Republic (CF). We ran 3 chains for 10,000,000 generations with trees being sampled every 1,000 generations. To infer phylogenetic relationships using maximum likelihood we used PhyML 3.0 [61].

### Adaptive Evolution Analyses

A common method to detect selection in protein coding genes is to estimate ω, the non-synonymous to synonymous rate ratio model [62], but this method is highly conservative and biased against detecting positive selection when a select few amino acid changes may result in adaptive changes. Due to the conserved nature of the mitochondrial genome, we used the algorithm implemented in TreeSAAP (Selection on Amino Acid Properties) [63] to identify significant amino acid changes among the members of Elephantidae. TreeSAAP compares the distribution of observed changes inferred from a phylogenetic tree with the expected random distribution of changes under neutral conditions. To test for significant amino acid changes in our dataset, we analyzed the phylogenetic tree for each of the 13 protein coding genes separately. TreeSAAP utilizes a sliding window to analyze the magnitude of change for 31 physicochemical properties of amino acids and rates those substitutions on a scale of 1 (most conservative) to 8 (most radical). A significant positive z-score for any of the physicochemical properties included in the analysis indicates more non-synonymous substitutions than are expected under neutral conditions, suggesting positive selection. We included all 31 physicochemical properties, set our sliding window equal to 15 codons, and considered only the most radical amino acid substitutions (categories 7–8, p≤0.001) that are expected to be linked to changes in function.

### Protein Structure Prediction and Analysis

Complex I is a large assembly consisting of seven mtDNA-encoded subunits, which are covered by one or two structural templates. Due to relatively low sequence-identities (18–42%, Table S3) between the sequences of the constituting protein subunits and their structural templates, we used a hybrid comparative approach to model the structure of the overall complex.

First, the protein sequences of the individual subunits for *L. africana* were aligned with the corresponding sequences of homologous subunits from both template structures, *T. thermophilus* and *E.coli*. MODELLER [64] was used to predict the tertiary structure for the mtDNA-encoded individual subunits (*ND1*, *ND2*, *ND3*, *ND4*, *ND4L*, *ND5*, *ND6*) of complex I in the African elephant. Second, we used Chimera [65] molecular structure visualization software to generate the overall structure of the savanna elephant complex I by structurally aligning individual subunits against complex I templates from *T. thermophilus*. Third, FoldX [66] structure refinement software was used to refine the modeled complex I by adjusting side chains to result in lower free energy levels, thus creating a more stable structure. Finally, to assess the quality of the modeled complex structure, we structurally aligned the model of complex I with each template structure to measure the RMSD value and the TM-score in TM-align [67]. The RMSD value represents the average deviation between the corresponding residues of two proteins. Smaller values indicate higher similarity between structures, and values increase as the length of the protein chain increases. Similarly, the TM-score assesses the topological similarity between two protein structures and produces an output between [0,1] with higher values indicating better models [68].

Once we modeled complex I for the African elephant, we calculated relative ASA values for each residue identified to be under positive selection using NACCESS [69] and determined whether the residues were located at chain-chain binding sites with FoldX (Table 2). ASA values represent the area of the residue that is in contact with the solvent and is used to distinguish the protein surface from the interior [32].

## Supporting Information

Table S1 **List of primer sequences used in this study, and the region they amplified in the forest elephant mitochondrial genome.**
(DOCX)

Table S2 **The model of evolution used for each partition for phylogenetic analysis as determined by FindModel.**
(DOCX)

Table S3 **Values representing percent sequence identity and coverage between the African elephant and two structural.**
(DOCX)
