# Supplementary material for: Evidence of Positive Selection in Mitochondrial Complexes I and V of the African Elephant
Source: PLoS One. 2014 Apr 2;9(4):e92587. doi: 10.1371/journal.pone.0092587 (PMC3973626; doi:10.1371/journal.pone.0092587)
Supplement: Table S1 — List of primer sequences used in this study, and the region they amplified in the forest elephant mitochondrial genome. (DOCX) [file pone.0092587.s001.docx]

| **Primer Name** | **Forward Sequence** | **Primer Name** | **Reverse Sequence** | **Bases covered**  **(incl. primers)** |
| --- | --- | --- | --- | --- |
| MT1F | CACCATGCATATCACCTCCA | MT1R | GCCATAGCTGAATCACAGCA | 15732-16023 |
| MT2F | ATTTTGGGGATGCTGTGATT | MT2R | TGTGTGTACGCTGGGAATTT | 15993-16166 |
| MT3F | AAATTCCCAGCGTACACACATA | MT3R | GGGGTTTGAAGAGATAGTTACA | 16149-16694 (incl. VNTR) |
| MT4F | CAAACCCCAAAAGCAGGACTAT | MT4R | GCTTGATGCCAGCTCTCTTT | 16744-16950, 0-187 |
| MT5F | AAAGAGAGCTGGCATCAAGC | MT5R | TCTCTGGCGGATAGCTTTGT | 168-567 |
| MT6F | ACAAAGCTATCCGCCAGAGA | MT6R | GCTTCATGGCCTTCAAT | 571-905 |
| MT7F | GTGGTTGAAGGCGGATTTAG | MT7R | CATCATTCCCTTGCGGTACT | 841-1233 |
| MT8F | TCAAAACATTCACTTACCAAAAAG | MT8R | AAAGAGCTGTACCCCTTTTGAA | 1128-1578 |
| MT9F | CCACAAAAACAAAATAATTCCAA | MT9R | TGCCTGTGTTGGGTTAACAA | 1490-1871 |
| MT10F | GGATGCCCGCTGATAGTTA | MT10R | GAGGTCACCCCAACCAAAAT | 1812-2260 |
| MT11F | CGAGAAGACCCTATGGAGCTTA | MT11R | TTTAGCTGGAGGCGTCTTAGT | 2152-2611 |
| MT12F | TCCAGTACGAAAGGACAGAAAA | MT12R | GAGGATATGGTATTGGAAGAGGA | 2553-3039 |
| MT13F | GCCCCAATTCTAGCCCTAAC | MT13R | TGATGATGTTAGCCCTAAC | 2942-3449 |
| MT14F | GAATACTCAGCTGGCCCTTTC | MT14R | CGTGGTGTAATTGGTAGCACTG | 3382-3830 |
| MT15F | CGAACCTAAACTCGAGAATTCAA | MT15R | TGGAACTCAGAAGTGGAATGG | 3780-4255 |
| MT16F | AACCAACCCTGTAGCATCAAA | MT16R | TGGTTATTGCAGGTGTTTTGT | 4174-4631 |
| MT17F | TTCACAACTTCAATATCATCCCTAA | MT17R | GCGTCTGATTTGCATTCAGTT | 4574-5069 |
| MT18F | CCTGAAATAAGGACTGCAAGA | MT18R | CCAAGAAGAGAGCCTGGTTG | 5014-5452 |
| MT19F | TTATTTGGTGCTTGAGCTGGT | MT19R | GCTGGAGGTTTTATGTTGATGA | 5392-5856 |
| MT20F | CCTTGCAGGAGTATCCTCTATTCT | MT20R | CGCCAGTTGGAATAGCAATAA | 5773-6294 |
| MT21F | GGCATAGACGTTGACACTCG | MT21R | TTCATGCAGTATAGGCATCTGG | 6218-6683 |
| MT22F | TTCCCCAACACTTTCTTGGA | MT22R | GGTGTGGTCATGGAAGTGAA | 6600-7103 |
| MT23F | ACAATTGGGGTTCCAAGATG | MT23R | ACAGCCCATGAATGGAGAAC | 7037-7527 |
| MT24F | CCTACAGACCTACCAGTTCGAG | MT24R | TCTTTCAAGGGAAAACACATGA | 7455-7932 |
| MT25F | TCGCCTTCTTTTCCCAATCT | MT25R | AGGTGTTCCTTGTGGGAGAA | 7871-8336 |
| MT26F | GTTGTCCTGGGTTTCCGATA | MT26R | AGCAGGAGGGTTACAGAGTGA | 8250-8735 |
| MT27F | CCCTACTTATAACATCCGGCCTA | MT27R | TCGGAGATTGTAAAGGATGC | 8675-9173 |
| MT28F | GGAGTCTACTTCACACTTCTCCAA | MT28R | AACGAGCAGAGCCTACTGGA | 9112-9603 |
| MTAF | GGGCCTCATTTCAGACTCAG | MTAR | GAGGCTATTTGACCAATAATGATG | 14778-15097 |
| MTBF | CACACACATTGGACGAAACA | MTBR | AATGGTATAGTACGGGTGAAAGG | 14436-14829 |
| MTCF | AAAACAATATACATCATTACTTCCACA | MTCR | TGCCGGTATTTCAAGTTTCC | 14057-14500 |
| MTDF | AGACGCTCCCCCAGAATAAT | MTDR | CAACGATGGTTTTTCAGATCA | 13642-14127 |
| MTEF | ATAGCCCTGGCTGTAACCTT | MTER | TCATGGTTCTTGGTGAAGAGG | 13174-13723 |
| MMFF | CCTCTGGCTCTATCATCCACA | MTFR | AAGTTCTGTTGCTACCGTAAATCC | 12765-13252 |
| MTGF | CCAACCCCAAGAACACTACA | MTGR | GTCCTCCTATTTTGCGGATG | 12336-12824 |
| MTHF | CACTGAATAACAATCCACACCCTA | MTHR | TGAGGCCAGGAGAAGACCTA | 11939-12400 |
| MTIF | CCTTTCCCTAAACCCCAAGA | MTIR | AGGGAAAAGAAGTCTAATTTAAAGC | 11502-12016 |
| MTJF | TTTCATGGGAGCACTTACCC | MTJR | TGTTTTGGTTAAACTATGTCTGCAA | 11088-11615 |
| MTKF | CCATACCTAATCAAACCCATTGA | MTKR | TTGAATTTGCTAGACAGAAAAGTATTG | 10690-11179 |
| MTLF | AGCCTAAACCTCTCCCCAGA | MTLR | GGTGGAGACCATATAGAGGTATTTTT | 10298-10804 |
| MTMF | GCCTTCGTTATTTCACTCATTF | MTMR | CAAATAGTAAGGATTAGTAGTGGGGTA | 9929-10376 |
| MTNF | TTCTAATCGCATTTTGACTACCC | MTNR | CGAAGATATTAGGTGAGAGCGGTA | 9539-9961 |
| MTYF | ACCACTATGTAACTATCTCTTCAAACC | MTYR | TGATTTGCTTTCACCCCTATG | 16664-16838 |
| MTZF | GCGTCCTAGCCCTACTCCTA | MTZR | GGTAGTTTTCGATTACTCCTGCAA | 15020-15277 |
| MDL 3 | Fernando et al.[^1^](#_ENREF_1) | MDL 5 | Fernando et al.[^1^](#_ENREF_1) | 15164-15803 |

1 Fernando, P., Pfrender, M. E., Encalada, S. E. & Lande, R. (2000) Mitochondrial DNA variation, phylogeography and population structure of the Asian elephant. Heredity 84: 362-372.
