# Supplementary material for: Evidence of Positive Selection in Mitochondrial Complexes I and V of the African Elephant
Source: PLoS One. 2014 Apr 2;9(4):e92587. doi: 10.1371/journal.pone.0092587 (PMC3973626; doi:10.1371/journal.pone.0092587)
Supplement: Table S2 — The model of evolution used for each partition for phylogenetic analysis as determined by FindModel. (DOCX) [file pone.0092587.s002.docx]

| **Partition** | **Model of Evolution** |
| --- | --- |
| ATP6 | HKY |
| ATP8 | HKY |
| COX I | GTR + G |
| COX II | HKY |
| COX III | GTR + G |
| CYT B | HKY + G |
| ND1 | GTR + G |
| ND2 | GTR + G |
| ND3 | HKY + G |
| ND4 | HKY + G |
| ND4L | HKY + G |
| ND5 | GTR + G |
| ND6 | GTR + G |
| tRNAs | GTR + G |
| rRNAs | GTR + G |
