# Supplementary material for: Evidence of Positive Selection in Mitochondrial Complexes I and V of the African Elephant
Source: PLoS One. 2014 Apr 2;9(4):e92587. doi: 10.1371/journal.pone.0092587 (PMC3973626; doi:10.1371/journal.pone.0092587)
Supplement: Table S3 — Values representing percent sequence identity and coverage between the African elephant and two structural. (DOCX) [file pone.0092587.s003.docx]

| **Subunits** | ***E. coli* template** | **Sequence Identity (%)** | **Coverage (%)** | ***T. thermophilus* template** | **Sequence Identity (%)** | **Coverage (%)** |
| --- | --- | --- | --- | --- | --- | --- |
| ND1 | - | - | - | 4HE8 chain H | 41 | 93 |
| ND2 | 3RKO chain N | 23 | 97 | 4HE8 chain N | 29 | 61 |
| ND3 | 3RKO chain A | 34 | 93 | 4HE8 chain A | 37 | 69 |
| ND4 | 3RKO chain M | 31 | 83 | 4HE8 chain M | 31 | 52 |
| ND4L | 3RKO chain K | 27 | 99 | 4HE8 chain K | 29 | 99 |
| ND5 | 3RKO chain L | 39 | 70 | 4HE8 chain L | 42 | 63 |
| ND6 | 3RKO chain J | 19 | 97 | 4HE8 chain J | 18 | 96 |
